# Neuroprotective effects of Danshensu on rotenone-induced Parkinson’s disease models in vitro and in vivo

**DOI:** 10.1186/s12906-019-2738-7

**Published:** 2020-01-23

**Authors:** Tian Wang, Cuiting Li, Bing Han, Zhenhua Wang, Xiaoyu Meng, Leiming Zhang, Jie He, Fenghua Fu

**Affiliations:** 1 School of Pharmacy, Key Laboratory of Molecular Pharmacology and Drug Evaluation (Yantai University), Ministry of Education, Collaborative Innovation Center of Advanced Drug Delivery System and Biotech Drugs in Universities of Shandong, Yantai University, Qingquan Road 30#, Yantai, Shandong 264005 People’s Republic of China; 2 Center of Mitochondria and Healthy Aging, School of Life Science, Yantai University, Qingquan Road 30#, Yantai, Shandong 264005 People’s Republic of China

**Keywords:** Parkinson’s disease, Danshensu, Rotenone, Glutathione

## Abstract

**Background:**

Danshensu is an active constituent in the extracts of Danshen which is a traditional Chinese medical herb. Rotenone inhibits complex I of the mitochondrial electron transport chain in dopaminergic neurons leading to glutathione (GSH) level reduction and oxidative stress. The aim of this study is to investigate neuroprotective effects of Danshensu on rotenone-induced Parkinson’s disease (PD) in vitro and in vivo.

**Methods:**

In vitro*,* SH-SY5Y human neuroblastoma cell line was pretreated with Danshensu and challenged with rotenone. Then the reactive oxygen species (ROS) production was assayed. In vivo, male C57BL/6 mice were intragastrically administered with Danshensu (15, 30, or 60 mg/kg), followed by oral administration with rotenone at a dose of 30 mg/kg. Pole and rotarod tests were carried out at 28 d to observe the effects of Danshensu on PD.

**Results:**

Danshensu repressed ROS generation and therefore attenuated the rotenone-induced injury in SH-SY5Y cells. Danshensu improved motor dysfunction induced by rotenone, accompanied with reducing MDA content and increasing GSH level in striatum. Danshensu increased the number of TH positive neurons, the expression of TH and the dopamine contents. The expressions of p-PI3K, p-AKT, Nrf2, hemeoxygenase (HO-1), glutathione cysteine ligase regulatory subunit (GCLC), glutathione cysteine ligase modulatory subunit (GCLM) were significantly increased and the expression of Keap1 was decreased in Danshensu groups.

**Conclusions:**

The neuroprotective effects of Danshensu on rotenone-induced PD are attributed to the anti-oxidative properties by activating PI3K/AKT/Nrf2 pathway and increasing Nrf2-induced expression of HO-1, GCLC, and GCLM, at least in part.

## Background

Parkinson disease (PD) is the second most common neurodegenerative disorder after Alzheimer’s disease. The clinical symptoms of PD include tremor, rigidity, postural instability, bradykinesia and akinesia [1]. The histopathological characteristics of PD are the selective and progressive loss of dopaminergic neurons in the substantia nigra followed by a profound loss of dopamine in the striatum. To date, the etiology of PD is still unclear. Previous studies report that environmental, genetic and stochastic factors contribute to the development and the progression of PD [2]. The etiology of PD is associated with the factors as follows: oxidative stress, neuroinflammation, toxic factors, genomic factors, and so on [3]. Biochemical and cellular abnormalities have been identified in the brains of PD patients including oxidative stress, free radical-mediated damage, inflammatory changes, and mitochondrial dysfunction [4]. Oxidative stress is a core contributor to the initiation and progression of PD [5]. Oxidative stress accompanied by protein aggregation, cell cycle reactivation, apoptosis and mitochondrial dysfunction are found in PD [6]. Oxidative stress with decreases of activities of glutathione peroxidase, catalase and a reduction of glutathione (GSH) has also been observed in PD patients [7]. It has been reported that GSH level is reduced and GSSG level is elevated in the substantia nigra of PD patients. The altered GSH/GSSG ratio in the substantia nigra of PD indicates that oxidative stress plays a key role in the pathogenesis of nigral cell death in PD [8]. In post-mortem brains of PD patients, lipid peroxidation products are 10-fold higher when compared with other brain regions and age-matched controls [9]. Therefore, control and regulation of oxidative stress may be a promising therapeutic approach for the PD patients.

The current treatments of PD aim to increase the dopamine level in striatum to ameliorate the associated motor deficits. These include the precursor of dopamine (levodopa), agonists of dopamine (pramipexole, ropinirole, rotigotine, and lisuride), MAO-B inhibitors (selegiline, rasagiline) [10]. However, these approaches do not represent a long-term solution, as each loses efficacy as dopaminergic neurodegeneration progresses over time. The unsatisfactory effects of conventional antiparkinsonian drugs have prompted the search for novel alternatives. Recent researches have identified that plants show a medicinal property against PD. Danshen is a traditional Chinese medical herb. It is the dry root and rhizome of *Salvia miltiorrhiza* Bunge. In China, Danshen has been widely used to treat central nervous system diseases. Previous study shows that Tanshinone I represses the expression of pro-inflammatory genes in activated microglia. Tanshinone I also prevents dopaminergic neurodegeneration in an animal model of PD [11]. Tanshinone IIA inhibits the loss of dopaminergic neurons in substantia nigra. These findings indicate that Tanshinone possess the anti-inflammatory and anti-oxidative properties, and thereore may have the therapeutic value in the treatment of PD [12]. Salvianolic acid and Danshensu are the water-soluble constituents of Danshen. It reports that Salvianolic acid B attenuates 6-hydroxydopamine-induced apoptosis through inhibiting oxidative stress in SH-SY5Y cells [13]. The results from our laboratory show that Danshensu ameliorates the cognitive impairment of diabetic mice by attenuating neuroinflammation [14]. Danshensu also attenuates the reactive oxygen species (ROS) production by regulating PI3K/AKT/HO-1 signaling pathway in 6-OHDA-induced mice [15]. In the current study, we will investigate whether Danshensu has a neuroprotective effect on rotenone-induced PD models in vitro and in vivo.

## Methods

### Drug and chemical agents

Danshensu (Purity 99.6%) was from Shandong Luye Pharmaceutical Co., Ltd. (Yantai, China). Rotenone, dimethylsulfoxide (DMSO), streptomycin/penicillin, dopamine, DCFH-DA, and GSH were purchased from Sigma Chemical Company (St. Louis, MO, USA). Dulbecco’s modified Eagle’s medium (DMEM) was from Gibco Company (Grand Island, NY, USA). Fetal bovine serum (FBS) was provided by Zhejiang Tianhang Biotechnology Co., Ltd. (Hangzhou, China).

### Cell culture

The SH-SY5Y human dopaminergic neuroblastoma cell line was purchased from the Shanghai Cell Bank (Shanghai, China). The cells were cultured in DMEM supplemented with 10% FBS and streptomycin/penicillin (100 U/ml) at 37 °C in an atmosphere containing 5% CO_2_. Cell culture medium was changed for every 2 days.

### Determination of cell viability by MTT assay

SH-SY5Y cells were seeded in 96-well plates (1 × 10^4^ cells/well) and incubated overnight. Rotenone were freshly dissolved in DMSO and added to the cells at a final concentration of 0.5% (v/v) DMSO for each experiment. Danshensu dissolved in DMEM was added at 2 h prior to the rotenone exposure. To determine the safety of Danshensu and the toxicity of rotenone, cells were treated with different concentrations of Danshensu (0.1 μM, 1 μM and 10 μM) or rotenone (10 nM, 50 nM, 100 nM, 200 nM, and 400 nM) for 24 h. To evaluate the neuroprotective effect of Danshensu, SH-SY5Y cells were pretreated with Danshensu (0.1 μM, 1 μM and 10 μM) for 2 h and then challenged with rotenone (100 nM) for 24 h. The cells were incubated with 5 mg/mL MTT for 4 h at 37 °C in the dark. The medium was removed carefully after the incubation of MTT. The formazan crystals were dissolved in 200 μL DMSO and the absorbance of formazan reduction product was measured by microplate reader (Bio-Tek, USA) at 595 nm. Cell viability was expressed as values relative to the control with a control value of 100%.

### Evaluation of ROS by flow cytometry

Intracellular ROS levels were evaluated using DCFH-DA as a fluorescent probe. Briefly, SH-SY5Y cells (1 × 10^5^ cells/well in 6-well plates) were pretreated with Danshensu (1 μM) for 2 h and then were challenged with rotenone (100 nM) for 1 h or 6 h. The supernatant was removed and the cells were incubated with 1 mL DCFH-DA (10 μM, dissolved in serum-free DMEM) for 20 min at 37 °C in dark. Thereafter, the cells were rinsed twice with PBS and were resuspended with 1 mL PBS. The ROS levels were analyzed using a flow cytometry (ACEA Biosciences, USA) and were expressed as values relative to the control.

### Animals and treatments

Twelve-week-old male C57BL/6 mice (23–25 g) were purchased from Pengyue experimental animal company (Jinan, China). Mice were acclimated and maintained at 23 ± 1 °C under 12 h light/dark cycles. They were group-housed in cages and had free access to water and food. All animal experiments were carried out in accordance with the National Institutes of Health Guide for the Care and Use of Laboratory Animals (publication 86–23, revised in 1986) and the Committee of Yantai University for the Care and Use of Laboratory Animals (Approval number, YTU20140803). Mice (100) were randomly divided into 5 groups with 20 animals in each group: control group, rotenone group, Danshensu (15 mg/kg) group, Danshensu (30 mg/kg) group, Danshensu (60 mg/kg) group. Rotenone and Danshensu were suspended in 0.5% CMC-Na. The mice in Danshensu groups were continually treated with Danshensu at a dose of 15, 30 or 60 mg/kg by intragastric route once a day for 28 days. One hour after Danshensu treatment, the mice were intragastrically administered with rotenone (30 mg/kg) or an equivalent volume of 0.5% CMC-Na (control group) daily for 28 consecutive days. Two hours after the last administration of rotenone or 0.5% CMC-Na, neurobehavioral functions of mice were evaluated by experimenters who were blinded to the experimental design.

### Pole test

Pole test was performed according to the previous method [16]. The mice were placed on a wooden pole (1 cm diameter, 50 cm long). The time that mice took to climb down to the floor was recorded. Each mouse was tested three trials and the average time was calculated for further analyses.

### Rotarod test

An accelerating rotarod device (Yiyan Technology Development Co., Ltd., Jinan, China) was used to evaluate the motor coordination and balance as previously described with minor modifications [17]. An observer who were blinded to the experimental design performed the testing. The acceleration settings were 5, 15, or 30 rpm, starting from the lowest acceleration. The average latencies to fall from the rotating rod during the testing periods were calculated for each mouse. Three repeated trials were investigated on the day of testing.

### MDA and GSH assays

Five mice of each group were anesthetized with isoflurane inhalation. When the mice were unconscious, the striatum of the brain was collected. The striatum was homogenized in 4 volumes of 0.1 mol/L ice phosphate buffer (pH 7.4). Then they were centrifuged at 10,000 *g* for 10 min at 4 °C. BCA protein assay kit was used to evaluate the total protein in the supernatant (Beyotime Institute of Biotechnology, Shanghai, China). MDA content in striatum was assayed according to the previous method [18]. GSH was measured according to the previous method [19]. Readings were taken at activation/emission wave lengths of 340/420 nm.

### TH immunohistochemistry

After behavioral tests, three mice were anesthetized with isoflurane inhalation. The unconscious mice were performed by transcranial perfusion with 4% paraformaldehyde. The brains were transferred into 30% sucrose at 4 °C for 72 h. Immunohistochemical staining of a series of 10-μm thick coronal sections was performed. After incubated with the anti-TH antibody (1:200) at 4 °C for 24 h, the sections were incubated with biotinylated secondary antibody for 1 h at 37 °C. Then they were incubated with streptavidin peroxidase for 1 h at 37 °C. With an Olympus microscope (IX-70, Olympus Corp. Japan), the total number of TH-positive cells on each section was counted for the region of substantia nigra by persons who were blind to the treatment. Cell counts were determined sixteen sections through substantia nigra corresponding to the Bregma − 2.90 mm to − 3.68 mm from rats. And four animals in each group were used for cell count. On each section, a 150 × 150 μm^2^ grid was randomly placed on the image.

### Analysis of dopamine, DOPAC and HVA

Six mice of each group were anaesthetized with isoflurane inhalation. The striatum was collected and then was homogenized in ice-cold 0.1 M HClO_4_ containing 0.01% EDTA. After centrifuged (14,000 g, 4 °C, 10 min), the concentration of dopamine and its metabolites homovanillic acid (HVA) and 3,4-dihydroxyphenylacetic acid (DOPAC) in supernatant were assayed by high-performance liquid chromatography with fluorescence. Concentrations of dopamine, HVA, and DOPAC were expressed as μg/g tissue weight.

### Western blot

According to the manufacturer’s instructions, the protein extracts of nucleus and cytoplasm (three mice in each group) were prepared using nuclear and cytoplasmic protein extraction kit (Beyotime Institute of Biotechnology, Shanghai, China). The total protein was assayed using BCA protein assay kit (Beyotime Institute of Biotechnology, Shanghai, China). Proteins (50 μg) were subjected to 8% sodium dodecyl sulfate-polyacrylamide gel electrophoresis. After blocked with 5% nonfat milk for 1 h, the membranes were incubated overnight at 4 °C with the primary antibodies for cytosolic fractions: rabbit anti-TH antibody (1:1000, Millipore Corporation), rabbit anti-phospho-PI3K (1:1000, Cell Signaling Technology), rabbit anti-PI3K (1:1000, Cell Signaling), rabbit anti-phospho-AKT (1:1000, Cell Signaling), rabbit anti-AKT (1:1000; Cell Signaling), anti-GCLC (1:1000, Abcam), anti-GCLM (1:2000, Abcam), anti-Heme Oxygenase 1 (1:2000, Abcam), anti-Keap1 (1 μg/ml, Abcam), rabbit anti-β-Actin (1:1000; Cell Signaling), and the primary antibodies for nuclear fractions: anti-Lamin B1 (0.1 μg/ml, Abcam), anti-Nrf2 (1:1000, Abcam). The membranes were incubated with the horseradish peroxidase-labeled secondary antibody (1:2000). Bands were investigated using the ECL detection reagents (Beyotime Institute of Biotechnology, Shanghai, China).

### Statistical analysis

All data were expressed as the Mean ± SEM. Analysis was performed Using GraphPad Prism software 6.0. The data were analyzed with One-way ANOVA followed by Tukey’s post hoc test. Statistical significance was defined as *P* < 0.05.

## Results

### Effect of Danshensu on rotenone-induced cytotoxicity

**Fig. 1:**
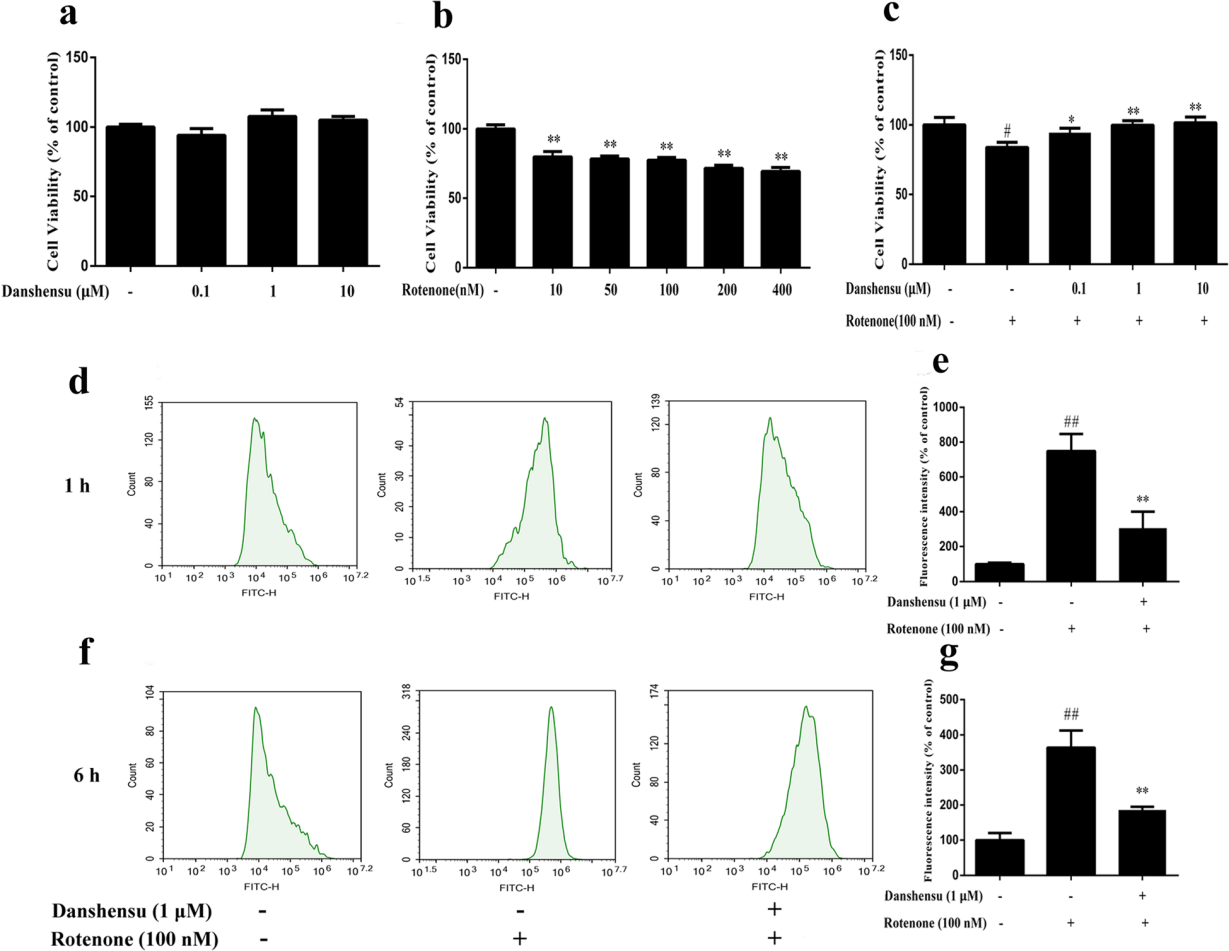
Danshensu attenuated the cytotoxicity and ROS generation induced by rotenone in SH-SY5Y cells. Data were expressed as the Mean ± SEM of three experiments. Statistical analyses were performed using One-way ANOVA followed by Tukey’s post hoc test. ^#^*P* < 0.05, ^##^*P* < 0.01 compared with the control group; ^*^P < 0.05, ^**^P < 0.01 compared with the rotenone group. **a** Effect of Danshensu on cell viability. **b** Effect of rotenone on cell viability. **c** Effect of Danshensu on rotenone-induced cytotoxicity. **d** Representative photographs of rotenone-induced ROS generation after treatment with rotenone for 1 h. **e** Bar graph of quantitative analysis of rotenone-induced ROS generation after treatment with rotenone for 1 h. **f** Representative photographs of rotenone-induced ROS generation after treatment with rotenone for 6 h. **g** Bar graph of quantitative analysis of rotenone-induced ROS generation after treatment with rotenone for 6 h

As shown in Fig. 1a, when exposed to Danshensu at concentrations of 10 μM or lower, the viability of SH-SY5Y cells had no significant difference as compared with the control cells. When exposed to rotenone for 24 h, a significant decrease of cell viability was observed with 10 nM and more significant toxicity was seen in 200 nM and 400 nM (Fig. 1b). Consequently, 100 nM of rotenone was used to induce cytotoxicity in the subsequent experiments. SH-SY5Y cells were pretreated with Danshensu for 2 h and then challenged with rotenone at concentration of 100 nM for 24 h. As shown in Fig. 1c, Danshensu (0.1 μM, 1 μM and 10 μM) significantly attenuated the cell toxicity induced by rotenone (*P =* 0.045, *P* = 0.0047, *P* = 0.0057, respectively) when compared with the rotenone group.

### Effect of Danshensu on rotenone-induced ROS generation

Figure 1d, e, f, g showed the effect of Danshensu on rotenone-induced ROS generation. The levels of intracellular ROS were significantly higher than the control group (100.0 ± 18.2%, 100.0 ± 20.1%) after treatment with rotenone for 1 h or 6 h (748.4 ± 196.5%, 363.9 ± 48.5%, *P* = 0.00059, *P* = 0.0024). However, pretreatment with Danshensu (1 μM) markedly decreased the rotenone-induced ROS generation (305.7 ± 189.1%, 185.9 ± 9.1%, *P* = 0.017, *P* = 0.011).

### Effect of Danshensu on the climbing time in rotenone-induced PD mice

**Fig. 2:**
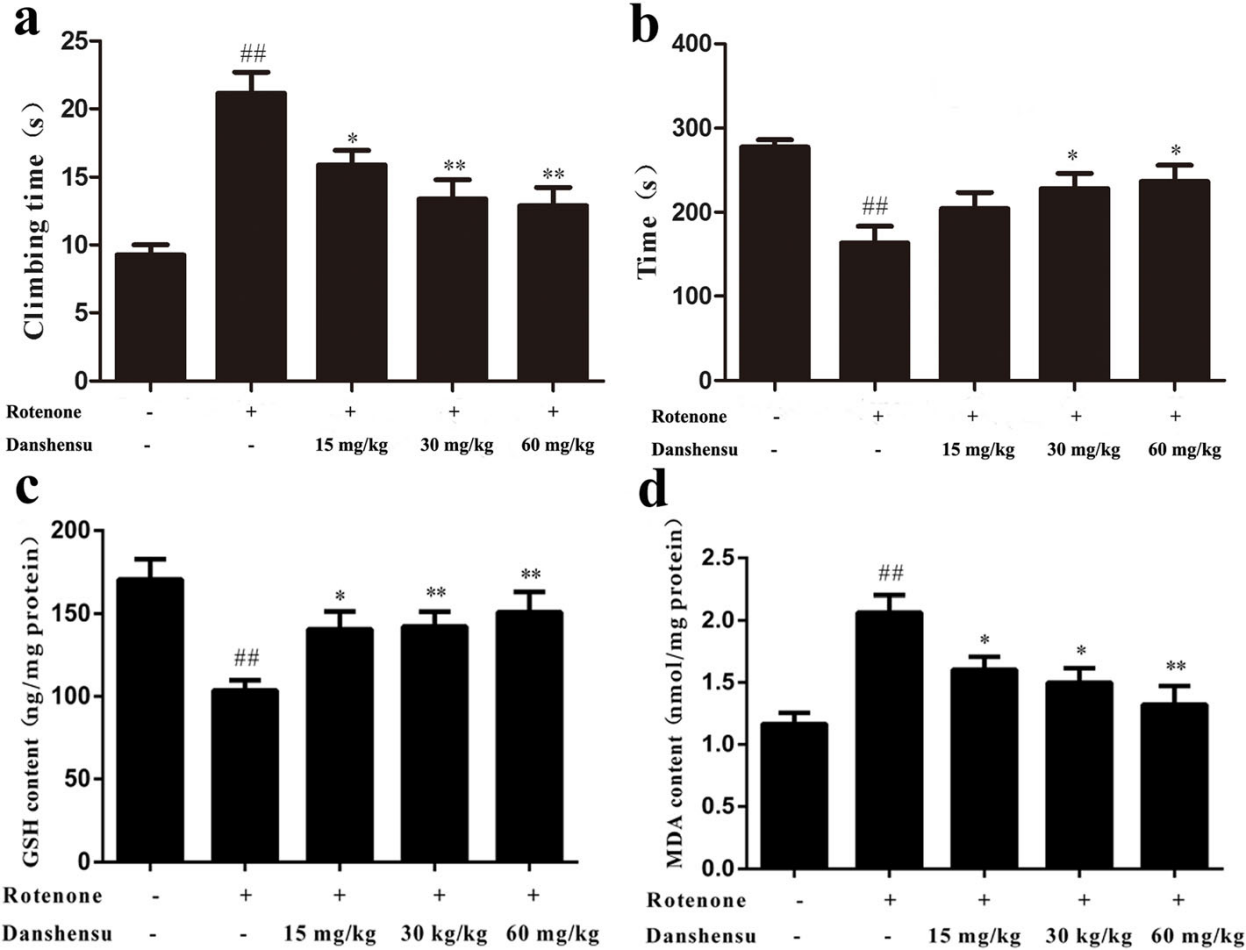
Effect of Danshensu on motor dysfunction, levels of GSH and MDA in the rotenone-induced PD mice. **a** Pole test, **b** Rotarod test, **c** GSH, **d** MDA. The data were expressed as the Mean ± SEM. Statistical analyses were performed using One-way ANOVA followed by Tukey’s post hoc test. ^#^*P* < 0.05, ^##^*P* < 0.01 compared with the control group; ^*^P < 0.05, ^**^P < 0.01 compared with the rotenone group

The climbing time in the control group was 9.3 ± 0.7 s. The climbing time of rotenone-induced mice PD model was significantly prolonged (21.1 ± 1.6 s, *P* = 2.42E-07). Danshensu (15, 30, and 60 mg/kg) treatment significantly shortened the climbing time (15.9 ± 1.1 s, 13.4 ± 1.4 s, and 12.9 ± 1.4 s, respectively; *P* = 0.01, *P =* 0.00097, *P =* 0.00041; Fig. 2a) compared with the rotenone group.

### Effect of Danshensu on the latency in rotenone-induced PD mice

Figure 2b showed the effect of Danshensu on the latency in rotarod test. The latency of the control group was 277.4 ± 8.4 s. Compared with the control group, the latency in the rotenone group (163.2 ± 20.1 s) decreased markedly (*P* = 1.98E-05). However, treatment with Danshensu at dose of 30, 60 mg/kg increased significantly the latency of PD mice (228.0 ± 18.1, 236.4 ± 19.5 s, *P* = 0.022, *P* = 0.013).

### Effect of Danshensu on the GSH level in rotenone-induced PD mice

The GSH level of the control group was 170.4 ± 27.6 ng/mg protein. Rotenone induced a significant decrease of GSH level (103.5 ± 14.1 ng/mg protein) in mice (*P* = 0.0013). Danshensu (15, 30, or 60 mg/kg) administration increased significantly the GSH level (140.5 ± 24.1, 142.2 ± 20.3, 150.8 ± 26.7 ng/mg protein, respectively, *P* = 0.018, *P =* 0.0080, *P =* 0.0081; Fig. 2c) compared with the rotenone group.

### Effect of Danshensu on the MDA content in rotenone-induced PD mice

Compared with the control group (1.16 ± 0.20 nmol/mg protein), rotenone caused a significant increase of MDA level in striatum (2.06 ± 0.31 nmol/mg protein, *P* = 0.00061). However, treatment with Danshensu at a dose of 15, 30, 60 mg/kg decreased markedly MDA content (1.60 ± 0.23, 1.49 ± 0.26, 1.32 ± 0.33 nmol/mg protein, respectively; *P* = 0.028, *P* = 0.014, *P* = 0.0065; Fig. 2d).

### Effect of Danshensu on the number of TH positive neurons and the TH expression in rotenone-induced PD mice

**Fig. 3:**
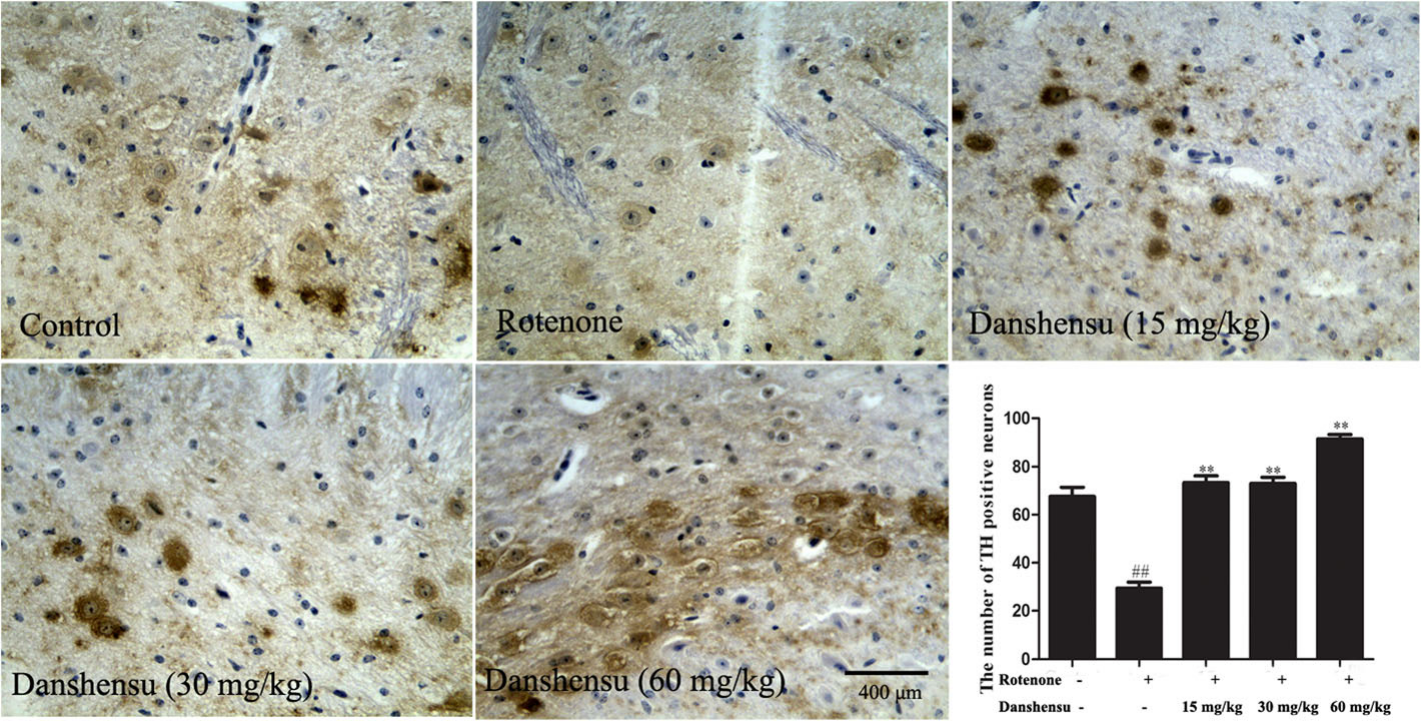
Effect of Danshensu on the number of TH positive neurons in rotenone-induced PD mice. Bar = 400 μm. ^##^*P* < 0.01 compared with the control group; ^**^P < 0.01 compared with the rotenone group

Figure 3 showed the representative microphotographs of TH positive neurons. The number of TH positive neurons in rotenone group was significantly decreased (*P* = 5.24E-28) compared with that of the control group. Treatment with Danshensu (15, 30, 60 mg/kg) for 28 days inhibited significantly the loss of the TH-positive neurons in rotenone-induced PD mice (*P* = 3.51E-25, *P* = 1.56E-26, *P* = 3.68E-30, respectively). The expression of TH was decreased in rotenone group when compared with that of the control group (*P* = 0.00035). The expression of TH in the striatum of Danshensu groups was markedly increased (*P* = 0.015, *P =* 0.0022, *P =* 7.43E-05; Fig. 6d, e).

### Effect of Danshensu on levels of dopamine, DOPAC and HVA in rotenone-induced PD mice

**Fig. 4:**
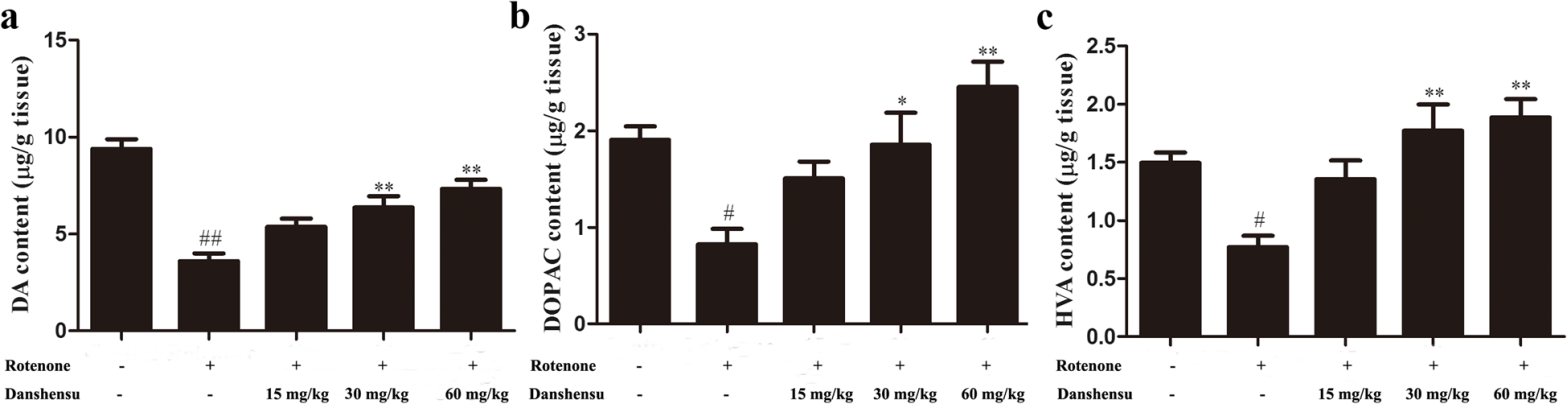
Effect of Danshensu on levels of dopamine (DA) (**a**), DOPAC (**b**) and HVA (**c**) in rotenone-induced PD mice. The data were expressed as the Mean ± SEM. Statistical analyses were performed using One-way ANOVA followed by Tukey’s post hoc test. ^#^*P* < 0.05, ^##^*P* < 0.01 compared with the control group; ^*^P < 0.05, ^**^P < 0.01 compared with the rotenone group

In Fig. 4a, b, c, the levels of dopamine, DOPAC and HVA in the striatum of the rotenone group decreased 62, 57 and 48%, respectively (*P* = 3.92E-06*, P* = 0.00049, *P* = 0.00030), compared with that of the control group. Compared with the rotenone group, Danshensu treatment resulted in significant increases of striatal dopamine (*P* = 0.012, *P* = 0.0026, *P* = 0.00014), DOPAC (*P* = 0.0162, *P* = 0.019, *P =* 0.00030) and HVA (*P* = 0.011, *P* = 0.0023, *P* = 0.00013) in PD mice.

### Effects of Danshensu on the expressions of proteins in PI3K/AKT/Nrf2 pathway in rotenone-induced PD mice

**Fig. 5:**
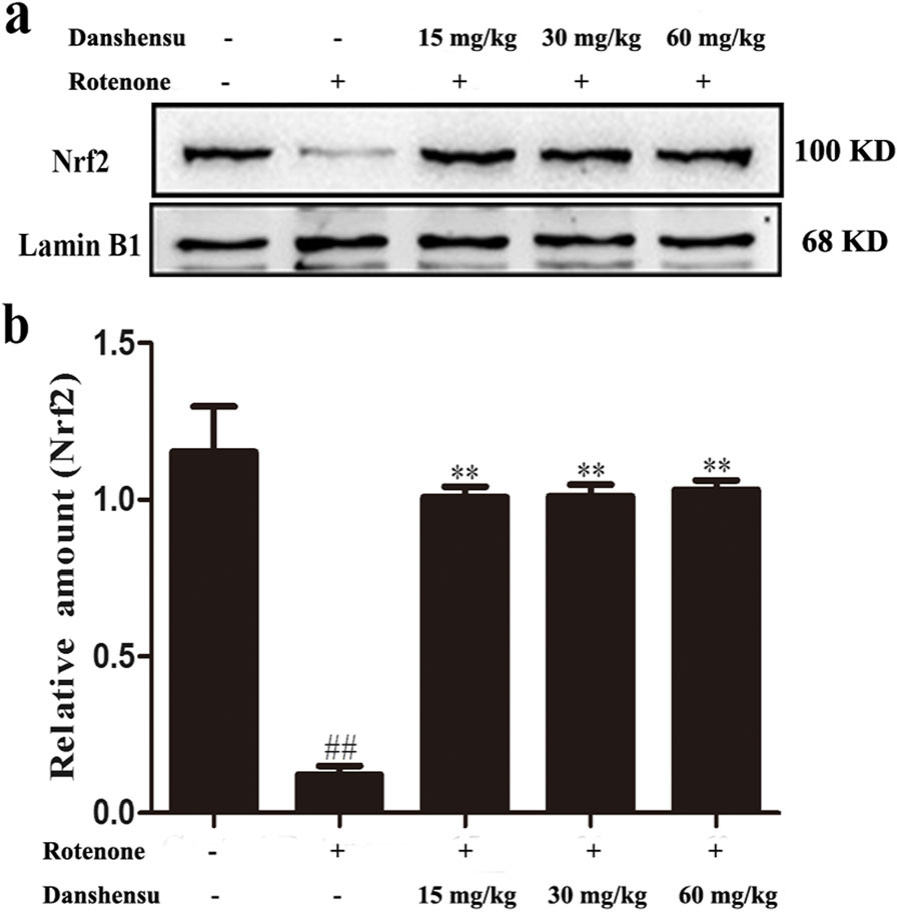
Effect of Danshensu on the expressions of nuclear translocation of Nrf2 in rotenone-induced PD mice. **a** showed representative photographs of Nrf2 in western blot measurement. **b** indicated bar graphs of Nrf2 expression in western blot measurement. The data were expressed as the Mean ± SEM. Statistical analyses were performed using One-way ANOVA followed by Tukey’s post hoc test. ^##^*P* < 0.01 compared with the control group; ^**^P < 0.01 compared with the rotenone group

**Fig. 6:**
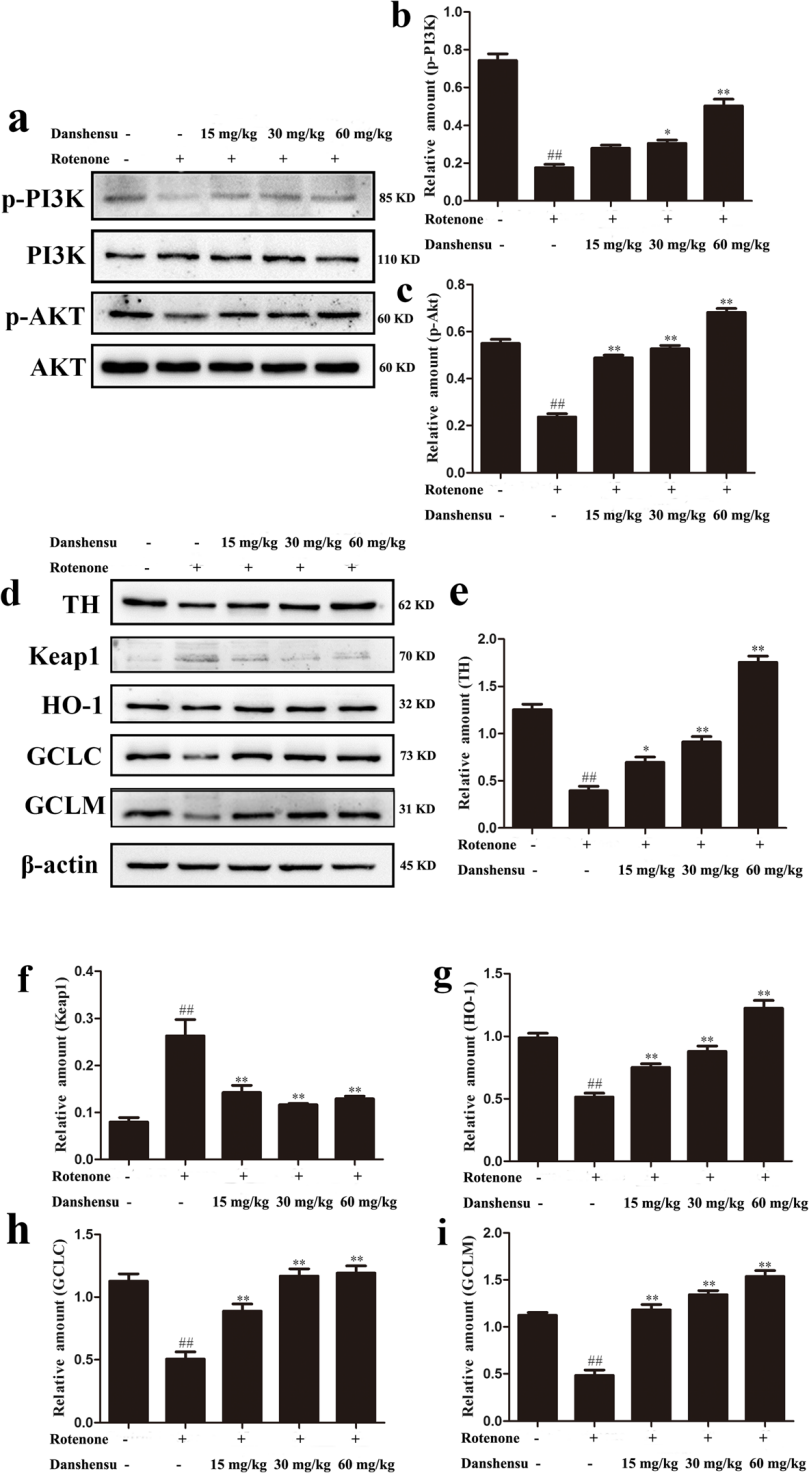
Effect of Danshensu on the expressions of nuclear translocation of Nrf2 in rotenone-induced PD mice. **a** showed representative photographs of Nrf2 in western blot measurement. **b** indicated bar graphs of Nrf2 expression in western blot measurement. The data were expressed as the Mean ± SEM. Statistical analyses were performed using One-way ANOVA followed by Tukey’s post hoc test. ^##^*P* < 0.01 compared with the control group; ^*^P < 0.05, ^**^P < 0.01 compared with the rotenone group. **a** Representative photographs of p-PI3K, PI3K, p-AKT, AKT in Western blot. **b** Bar graph of quantitative analysis of the expression of p-PI3K. **c** Bar graph of quantitative analysis of the expression of p-AKT. **d** Representative photographs of TH, Keap1, HO-1, GCLC, GCLM in Western blot. **e** Bar graph of quantitative analysis of the expression of TH. **f** Bar graph of quantitative analysis of the expression of Keap1. **g** Bar graph of quantitative analysis of the expression of HO-1. **h** Bar graph of quantitative analysis of the expression of GCLC. **i** Bar graph of quantitative analysis of the expression of GCLM

In this study, the expressions of proteins in PI3K/AKT/Nrf2 were assayed. The results showed that the expressions of nuclear Nrf2 (*P* = 0.00060), p-PI3K (*P* = 0.00013), p-AKT (*P* = 0.00016), HO-1 (*P* = 0.00068), GCLC (*P* = 0.0016), and GCLM (*P* = 0.00060) were markedly decreased. In contrast, the expression of Keap1 of rotenone group (*P* = 0.00074) was increased compared with that of the control group (Figs. 5 and 6). Danshensu treatment augmented the expressions of Nrf2 (*P* = 0.028, *P* = 0.014, *P =* 0.0065), p-PI3K (*P* = 0.052, *P* = 0.011, *P =* 0.0011), p-AKT (*P* = 0.00016, *P* = 0.00012, *P =* 3.46E-05), HO-1(*P* = 0.0053, *P* = 0.0053, *P =* 0.00053), GCLC (*P* = 0.0094, *P* = 0.0012, *P =* 0.0011), GCLM (*P* = 0.0010, *P* = 0.00029, *P =* 0.00025). Furthermore, Danshensu treatment also decreased the expression of Keap1 (*P* = 0.0063, *P* = 0.0011, *P =* 0.0018; Figs. 5 and 6).

## Discussion

Although the mechanisms of neurodegeneration in PD are not fully elucidated, excessive free-radical formation and oxidative stress may be related to the loss of dopaminergic neurons. It has been proposed that the dysfunction of mitochondrial complex I in the substantia nigra may be an important factor in the pathophysiology of PD. Being a mitochondrial complex I inhibitor, rotenone is widely used to evaluate the relationship of oxidative stress and the injury of dopaminergic neurons. This study showed that Danshensu attenuated the rotenone-induced toxicity and ROS generation in SH-SY5Y cells. Danshensu also improved the rotenone-induced motor dysfunction and reduced the ROS level in PD mice. Danshensu diminished partially the injury of the dopamine neurons in substantia nigra and the decrease of dopamine in the striatum. These findings suggested that the neuroprotective effects of Danshensu were related to regulating PI3K/AKT/Nrf2 pathway and inhibiting oxidative stress.

The results showed that 10 μM Danshensu had no effect on the cell viability and rotenone exposure caused a significant SH-SY5Y cell death and an increase of ROS generation. However, Danshensu protected SH-SY5Y cells against rotenone neurotoxicity as evidenced by enhancing cell viability and reducing ROS generation. Previous study indicates that rotenone causes dopaminergic neuronal loss in the substantia nigra region and reduces striatal dopamine levels, which leads to motor deficits in mice [20]. To evaluate the effects of Danshensu on the rotenone-induced motor dysfunction in PD model, pole and rotarod tests were performed. In the present study, Danshensu shortened the climbing time and increased the latency. These results demonstrated that Danshensu possessed neuroprotective effects that manifested as improvements of motor dysfunction in PD mice. In line with the results of in vitro, Danshensu also resulted in the increase of GSH content followed by the decrease of MDA level. These results suggested that the neuroprotective effects of Danshensu against rotenone toxicity are attributed to the anti-oxidative properties.

In the brains of PD patients, the dopamine concentrations of the substantia nigra and the striatum are decreased. Dopamine deficiency of the nigro-striatal system will cause movement disorders. Dopamine is catalyzed by catechol-O-methyltransferase and monoamine oxidase and then produce DOPAC and HVA. In brains, the levels of DOPAC and HVA are proportional to dopamine concentrations. TH is a rate-limiting enzyme for the biosynthesis of dopamine. More evidences suggest that TH plays a key role in the pathogenesis of PD [21]. In this study, we further examined the number of TH-positive dopaminergic neurons in the substantia nigra. And the expression of TH, the contents of dopamine, DOPAC and HVA in striatum were also observed. The results showed that Danshensu increased the number of TH-positive neurons and enhanced the expression of TH and the contents of dopamine, DOPAC, HVA. These results were consistent with the motor improvement of Danshensu observed in behavioral tests.

Oxidative stress has been proposed to play a pivotal role in the pathogenesis of PD [22]. A promising approach that focuses on inhibiting oxidative stress-related neurodegeneration is the activation of Nrf2 signaling pathway [23]. Nrf2, a transcription factor, plays a key role in attenuating the oxidant- induced injury. It is an important regulator in coordinating the expression of cytoprotective genes, including GCLC, GCLM, and HO-1 [24]. GCLC and GCLM are the rate-limiting enzymes in glutathione biosynthesis [25]. Keap1 inhibits the activation of Nrf2. Keap1 also facilitates the proteasomal degradation of Nrf2 [26]. Inhibition of Nrf2-mediated transcription augments a vulnerability of dopaminergic neurons to oxidative stress [27]. However, Nrf2 activation will exert a neuroprotective effect [28]. Previous studies indicated that the signaling pathways, including PI3K/AKT, mitogen activated protein kinase, and extracellular signal-regulated kinases [29], contributed to the ability of nuclear translocation and transcriptional activation of Nrf2 [30]. Especially, the PI3K/AKT signaling pathways play a pivotal role in regulating the expression of Nrf2-mediated downstream genes [31].

In this study, we observed that Danshensu activated the PI3K/AKT signaling pathway. There was also an increase of Nrf2 nuclear translocation accompanied by a significant decrease of Keap1 level in cytoplasm in Danshensu groups. Moreover, our data showed that the expressions of HO-1, GCLC, and GCLM were enhanced after Danshensu treatment. In line with the above findings, Danshensu also resulted in the increase of GSH content followed by the decrease of MDA level. These results suggested that the neuroprotective effects of Danshensu are attributed to the anti-oxidative properties by activating PI3K/AKT/Nrf2 pathway and increasing Nrf2-induced expression of HO-1, GCLC, and GCLM, at least in part.

## Conclusions

In conclusion, this study indicated that Danshensu possessed neuroprotective effects in rotenone-challenged SH-SY5Y cell and PD mice. Danshensu improved motor dysfunction in the PD animal model and the neuroprotective effects of Danshensu against rotenone toxicity are attributed to the anti-oxidative properties by activating PI3K/AKT/Nrf2 pathway and increasing expression of HO-1, GCLC, and GCLM, at least in part.

## Data Availability

The raw data and the full western blots of this study are included in the supplementary files.

## Abbreviations

CMC-Na: Sodium carboxymethylcellulose
DOPAC: 3,4-dihydroxyphenylacetic acid
GCLC: Glutathione cysteine ligase regulatory subunit
GCLM: Glutathione cysteine ligase modulatory subunit
GSH: Glutathione
HO-1: Hemeoxygenase
HVA: Homovanillic acid
MDA: Malondialdehyde
PD: Parkinson’s disease
ROS: Reactive oxygen species
TH: Tyrosine hydroxylase
